# Activation extending based on long-range dependencies for weakly supervised semantic segmentation

**DOI:** 10.1371/journal.pone.0288596

**Published:** 2023-11-21

**Authors:** Haipeng Liu, Yibo Zhao, Meng Wang, Meiyan Ma, Zhaoyu Chen

**Affiliations:** 1 Faculty of Information Engineering and Automation, Kunming University of Science and Technology, Kunming, China; 2 Yunnan Key Laboratory of Artificial Intelligence, Kunming University of Science and Technology, Kunming, China; Wuhan University of Science and Technology, CHINA

## Abstract

Weakly supervised semantic segmentation (WSSS) principally obtains pseudo-labels based on the class activation maps (CAM) to handle expensive annotation resources. However, CAM easily involves false and local activation due to the the lack of annotation information. This paper suggests weakly supervised learning as semantic information mining to extend object mask. We proposes a novel architecture to mining semantic information by modeling through long-range dependencies from in-sample and inter-sample. Considering the confusion caused by the long-range dependencies, the images are divided into blocks and carried out self-attention operation on the premise of fewer classes to obtain long-range dependencies, to reduce false predictions. Moreover, we perform global to local weighted self-supervised contrastive learning among image blocks, and the local activation of CAM is transferred to different foreground area. Experiments verified that superior semantic details and more reliable pseudo-labels are captured through these suggested modules. Experiments on PASCAL VOC 2012 demonstrated the proposed model achieves 76.6% and 77.4% mIoU in val and test sets, which is superior to the comparison baselines.

## Introduction

Semantic segmentation [1–3] can generate a regional mask containing semantic information for the input images. It has been widely used in various fields such as Medical imaging analysis [4], automatic driving [5], and Uav application [6]. However, most existing methods still rely on the manually labeled pixel-level label, which is hugely resource-consuming. In recent years, researchers are committed to the semantic segmentation method with semi-supervision [7], non-supervision [8] and weak supervision. Compared to semi-supervised methods, weakly supervised methods offer lower annotation costs, superior performance compared to unsupervised methods, and are better suited for semantic segmentation tasks [9], that is, to utilize cheaper annotations as supervisory information of the backbone networks, for instance, image-level classification labels [10, 11], scribbles [12], and bounding boxes [13], etc. These methods effectively reduce the implementation cost of this vision task. However, due to the lack of annotation information, this requires the model to discriminate the edge and shape of the object more finely. This paper’s method focuses on generating image-level classification labels through long-range dependencies between pixels.

**Fig 1:**
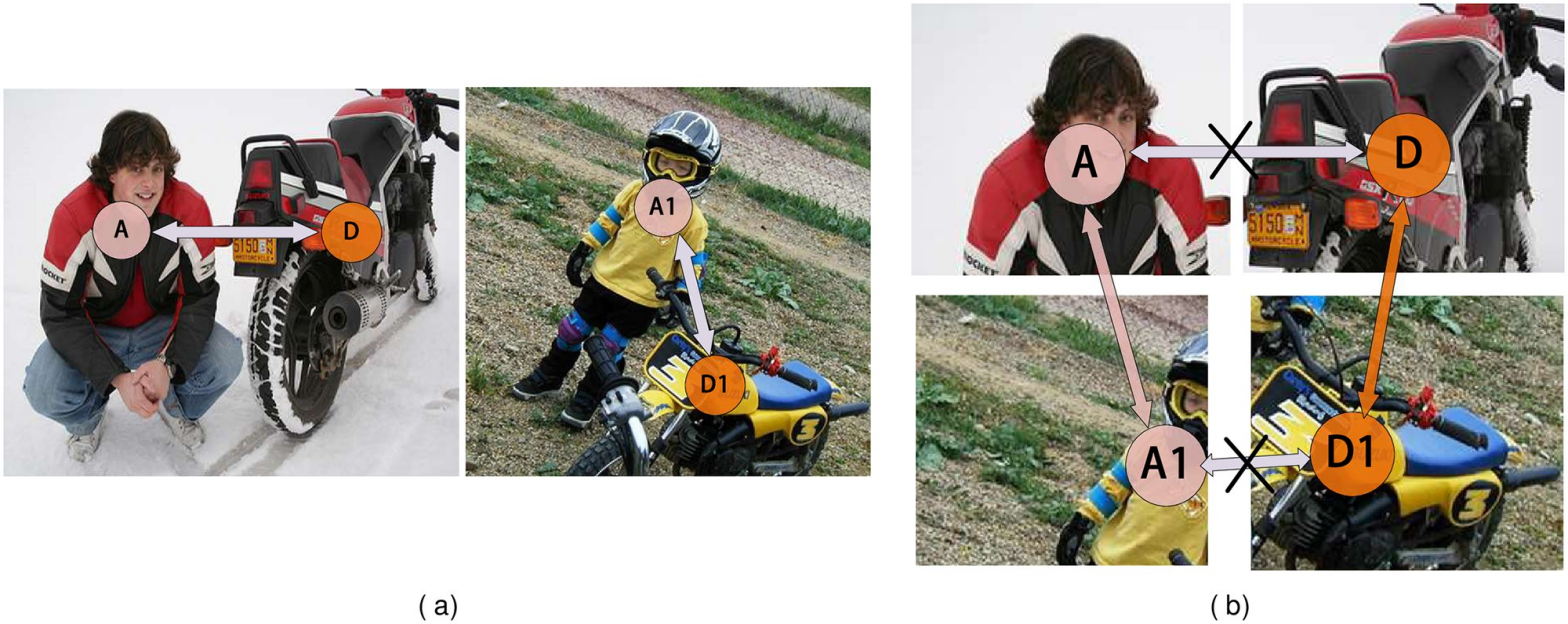
(a) Causal intervention in the sample caused by long-range dependencies. The same class is represented in the same color, where *A* ↔ *D* and *A*1 ↔ *D*1 represent false causal between different classes of regions. (b) Modeling of long-range dependencies between regional samples. The long-range dependences of the same classes in different samples(*A* ↔ *A*1,*D* ↔ *D*1) is established and the false causal between different classes is cut off. Each class captures long-range dependencies through pixel dependencies in a smaller area.

In the WSSS scene, the mostly schemes are suggested to extract practical information only by providing weaker labels than pixel-level labels [14], and convert the weak labels containing almost no object position information into image segmentation masks [10, 15]. Also, class-activated mapping (CAM) [16] is an effective solution to generate pixel-level pseudo-labels through image-level classification labels. However, due to the discriminant mode of the classifiers [17, 18], and these labels contain limited spatial details [19], that often leads to the local activation regions [20], and the segmented object boundaries easily involve false activation. They thus will cause different degrees of fragmentary masks [21]. A lot of recent work has refined the quality of CAM by mining more semantic and object location information from limited annotation information [10, 11, 18]. The success of these methods depends on the long-range dependencies [22] between pixels in an image. Long-range dependencies modeling can effectively improve the scene understanding ability of deep neural networks [23]. Still, these methods often use stacked convolution operations to obtain larger receptive fields to obtain this relationship [24]. Such repeated local operations make the computational complexity of the network too high. It is not conducive to network optimization [25]. As a non-local means operation, the self-attention mechanism [13] can calculate the correlation of elements at different spatial locations [26]. [27, 28] adopts self-attention mechanism to capture long-range dependencies to improve the prediction ability of CAM. However, these methods still have two drawbacks: (1) The long-range dependencies will mislead the image-level classification model to learn the false correlation between pixels and labels [29]. (2) These methods ignore the rich long-range dependencies between image samples [30]. Taking Fig 1 as an example, when the classification task is the goal, the correct classification of different classes benefits from their context, but in the segmentation tasks, this dependency is overemphasized, and the inter-pixel causal intervention [31] will make it difficult for CAM’s prediction to distinguish the boundaries. The lack of labeling information of pseudo-labels is the main reason for the performance gap between weakly and fully supervised models [32, 33]. How to establish the long-range dependencies between the same class between samples is also the main point of this paper.

According to the above, the bottleneck lies in how to mine more semantic information effectively and avoid information confusion caused by long-range dependencies to generate high-quality CAM. This paper formulates two novel modules to solve these difficulties. The first is the modified self-attention module [26], which is inspired by the puzzlecam [34] block method to carry out feature extraction on a smaller area, and explicitly cut off the similarity calculation between easily confused classes in the sample. This not only provides a kind of data enhancement for network training, but also provides richer and more accurate samples for subsequent contrastive learning. The second module is the foreground feature contrast based on cross-image analysis. It leverages existing feature information to enable pixel-level self-supervised contrast learning without negative samples. It can be used to strengthen relationships between similar prospects in different samples, and the loss is calculated by rank weight, which reduces the interference between different classes. To sum up, this paper has achieved the following points:

- We propose semantic mining to compensate the lack of annotation information in WSSS, modeling through long-range dependencies between in-sample and inter-sample, global and local, which narrows the gap between weak and full supervision;
- The proposed region self-attention module (RSA) calculates the correlation among pixels within a given sample area, using modified non-local self-attention to mitigates the information confusion caused by causal intervention and reduces false activations of CAM.
- The proposed cross-image contrast module (CFC) employs global and local weighting in contrastive learning. Reframe foreground features as positive samples, minimize the feature distance between samples of the same class. It effectively extends the local activation of CAM to the entire target area.
- Our proposed approach does not involve refinement of the CAM via additional networks, achieves 76.6% and 77.4% mIoU in val and test sets on PASCAL VOC 2012 and 43.8% mIoU in val set on MS COCO, the performance exhibited is of a superior nature.

## Related work

### Weakly supervised semantic segmentation

The strategies for pseudo-pixel label generation based on semantic information mining can be divided into region mining and cross-image mining. Among them, the region mining strategy [11, 26, 35] focuses on the pixel correlation of the single image, [36] drives the classification network to discover new and supplementary target regions sequentially by erasing the currently mined areas in an antagonistic manner, This approach also essentially breaks down the causal interference between pixels brought about by the long-range dependencies. [20] scheme was proposed to provide recalibration supervision for the CAM to some extent solve the CAM overactivation problems. SEAM [32] suggests a self-supervised equal-variable attention mechanism to narrow the gap between weak and complete supervision. Some studies have also explained CAM generation from a new perspective, such as causal reasoning, information bottleneck theory [37], and anti-resistant aggression [14]. However, these methods need to take advantage of the rich long-range dependencies between samples.

There are also methods for refining CAM based on mining semantic information cross-images [9, 38], SUN [39] proposed two neural co-attentions are incorporated into the classifier to capture cross-image semantic similarities and differences complimentarily. CIAN [33] suggested a cross-image attention module to learn activation mapping from two images containing objects of the same class under the guidance of saliency mapping. But these methods require additional data annotation. CCAM [30] proposes to generate a class-agnostic activation map for contrast learning as a background cue for CAM, but this approach requires additional classifier generation to be a class-agnostic activation map. Our approach is based on cross-image and region mining, and no more convolution modules or annotation data sets were added.

### Non-local self attention operation

In deep neural networks, convolution and cyclic operations deal with local space or time neighborhoods. Therefore, long-range dependencies can only be captured when these operations are repeatedly applied. The self-attention module can compute the response at a location by attending to all locations and taking their weighted average in the embedding space [26]. [27] integrate class-agnostic saliency priors into the self-attention mechanism and utilize class-specific attention cues as additional supervision. [40] propose an Unbiased self-attention learning segmentation network, which designs unbiased layers to guide the network to expand the discrimination field of CAM during training. [41] propose an edge-based self-attention mechanism to strengthen the nodule edge segmentation effect. Influenced by the self-attention mechanism of VIT [24], SEAM [32] introduces a pixel-dependent module (PCM) that captures contextual appearance information for each pixel and modifies the original CAM with a learned affinity attention map. [26] proposed a non-local operation to calculate the response of one location as a weighted sum of all location features, as a means of image information mining, to capture long-distance context relations. But essentially, context is an obfuscator that creates false causal interventions between pixels [29].

## Methodology

### Class activates mapping (CAM)

**Fig 2:**
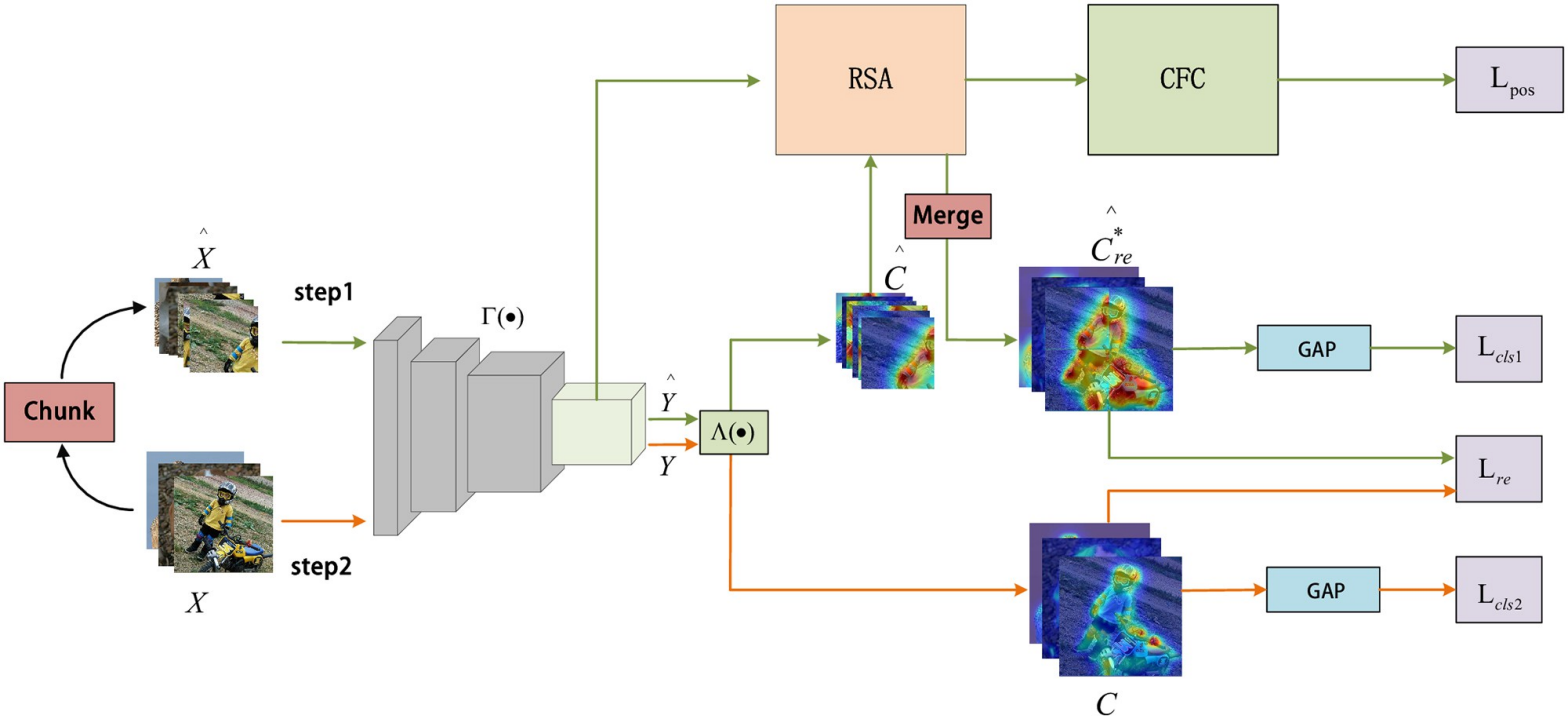
We propose a two-step network structure. CAMs in the figure result from the visualization of all the classes. The ${\hat{C}}^{*}$ generated by RSA was restored to the size of *C* by the Merge module after being corrected by the cross-image comparison module, *C* generated by two-step provides equivariant constraint supervision for the merged version of ${\hat{C}}_{re}^{*}$.

In this section, we describe the procedure for generating class activation maps (CAM) [27], CAM can show the distribution of contributions to classification on the original images. The process of the method in this paper is shown in Fig 2. Given a training set of images is defined as $X={\left\{x_{i}\right\}}_{i=1}^{Q}$, the generation of CAM is divided into two steps. Firstly, the input image *X* is uniformly divided into 2×2 non-overlapping image blocks $\hat{X}={\left\{{\hat{x}}_{i}\right\}}_{i=1}^{Q}$ through the Chunk module for step 1, where ${\hat{x}}_{i}=\left[{\hat{x}}_{i}^{\left(1\right)},{\hat{x}}_{i}^{\left(2\right)};{\hat{x}}_{i}^{\left(3\right)},{\hat{x}}_{i}^{\left(4\right)}\right]$, and CAM $\hat{C}={\left\{{\hat{c}}_{i}\right\}}_{i=1}^{Q}$ is later generated for each ${\hat{x}}_{i}^{\left(j\right)}$, where ${\hat{c}}_{i}=\left[{\hat{c}}_{i}^{\left(1\right)},{\hat{c}}_{i}^{\left(2\right)};{\hat{c}}_{i}^{\left(3\right)},{\hat{c}}_{i}^{\left(4\right)}\right]$. Secondly, the generation of CAM $C={\left\{c_{i}\right\}}_{i=1}^{Q}$ of step 2 is performed on the original size image *X* at the same time. The output feature maps of the last convolutional layer generated through the encoder Γ(⋅) is defined as $\hat{Y}={\left\{{\hat{y}}_{i}\right\}}_{i=1}^{Q}$ of step 1 and $Y={\left\{y_{i}\right\}}_{i=1}^{Q}\in R^{H\times W\times D}$ of step 2, where ${\hat{y}}_{i}=\left[{\hat{y}}_{i}^{\left(1\right)},{\hat{y}}_{i}^{\left(2\right)};{\hat{y}}_{i}^{\left(3\right)},{\hat{y}}_{i}^{\left(4\right)}\right]$, ${\hat{y}}_{i}^{\left(j\right)}\in R^{M\times N\times D}$ and $y_{i}^{\left(j\right)}\in R^{H\times W\times D}$, *D* represents the number of channels, and *M*×*N* and *H*×*W* represents the size. A fully connected layer Λ(⋅) with parameter $A\in R^{D\times G}$ is later used to retrieve the classification scores, where *G* is the number of classes. The prediction score of two steps for class *p* is:

$$\begin{array}{c} \left\{ \begin{array}{c} {\hat{S}}_{p}=\frac{1}{MN}\sum_{z=1}^{D}A_{p,z}\sum_{\hat{x}=1}^{M}\sum_{\hat{y}=1}^{N}{\hat{Y}}_{\hat{x},\hat{y},z}\\ S_{p}=\frac{1}{HW}\sum_{z=1}^{D}A_{p,z}\sum_{x=1}^{H}\sum_{y=1}^{W}Y_{x,y,z} \end{array}\right. \end{array}$$ (1)

where ${\hat{Y}}_{\hat{x},\hat{y},z}$, *Y*_*x*,*y*,*z*_ represents the activation of $\hat{Y}$ and *Y* at its spatial location $\hat{x},\hat{y}$ and *x*, *y* on the *z* channel. We generate CAM for two steps by a weighted linear sum of visual patterns at different spatial locations. This process is described as follows:

$$\begin{array}{c} \left\{ \begin{array}{c} {\hat{C}}_{p}=\sum_{z=1}^{D}A_{p,z}{\hat{Y}}_{z}\\ C_{p}=\sum_{z=1}^{D}A_{p,z}Y_{z} \end{array}\right. \end{array}$$ (2)

where ${\hat{C}}_{p}$ and *C*_*p*_ represent the CAM of class p, the CAM $\hat{C}$ and *C* for all classes is obtained by concatenating ${\hat{C}}_{p}$ and *C*_*p*_, the activation function *Relu* is then applied to $\hat{C}$ and *C* to mask irrelevant pixels to obtain the final visual version of CAM, as shown in Fig 2. It is worth mentioning that global average pooling can be applied to CAM in practice to obtain a vector of classification prediction scores for all classes, which is equivalent to the set of all class prediction scores in Eq 1.

CAMs can be used as initial seed regions for pseudo-labels [32]. As seen from the above methods, such CAM is based on the classification task as the target, so it will cause the CAM to be often limited to the area with higher classification prediction scores [20], which is unfavorable for our pixel-level segmentation task. Two kinds of modules are proposed to obtain more reasonable CAM prediction masks, which will be elaborated on in the next section.

### Regional self-attention module (RSA)

**Fig 3:**
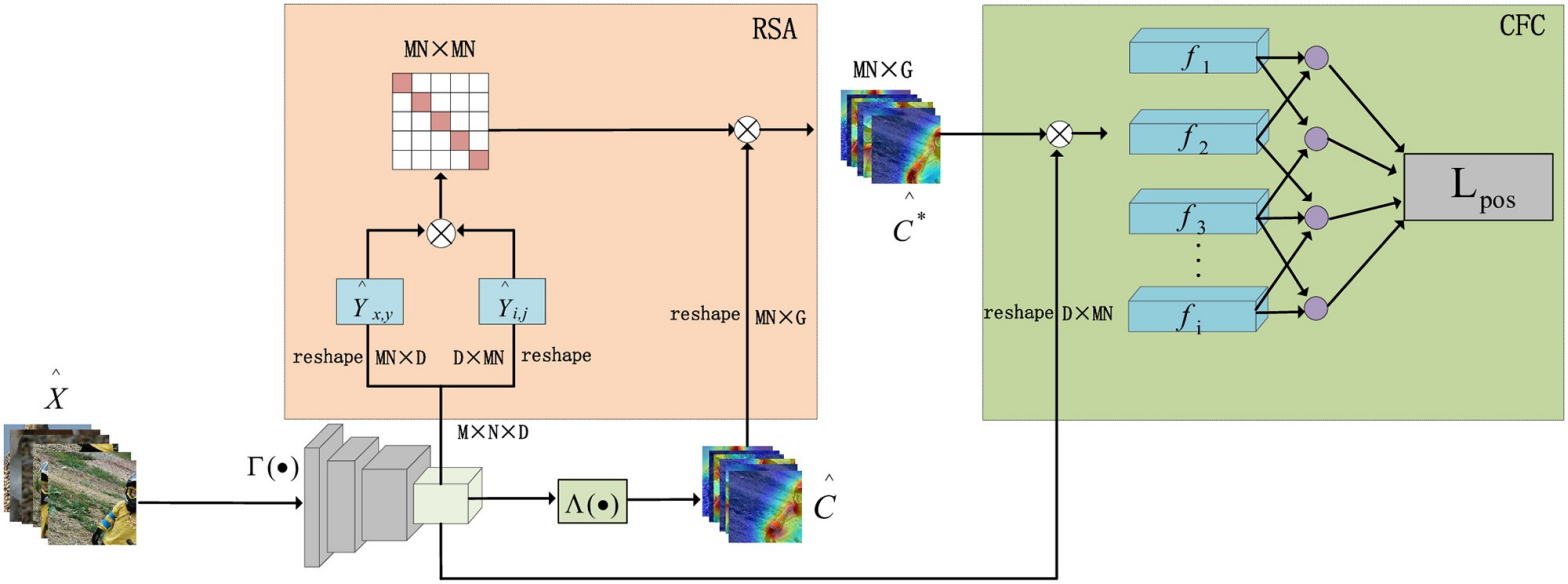
The two modules applied in step 1 of Fig 2 is described in detail. The reshape represents tensor size transformation, ⊗ Stands for matrix dot product operation. *RSA* module: The similarity matrix with the calculated size of *MN* × *MN* is normalized,The refined CAM ${\hat{C}}^{*}$ can be obtained by weighting the original CAM $\hat{C}$. *CFC* module:The foreground vector is formed by matrix multiplication of ${\hat{y}}_{i}$ and ${\hat{c}}_{i}^{*}$.

RSA is a module that captures contextual information and optimizes pixel-level prediction results. The detailed design of the proposed RSA module is shown in Fig 3. We improve the self-attention module proposed by [26]. The classical self-attention module given in this method is calculated as follows:

$$\begin{array}{c} C_{x,y,z}^{*}=\frac{1}{g(Y)}\sum_{i}^{H}\sum_{j}^{W}e^{\theta {\left(Y_{x,y,z}\right)}^{T}\phi \left(Y_{i,j,z}\right)}\varphi \left(C_{x,y,z}\right)+C_{x,y,z} \end{array}$$ (3)

in Eq 3, *C*_*x*,*y*,*z*_ and $C_{x,y,z}^{*}$ represent the original CAM and the modified CAM with spatial position *x*, *y* on the *z* channel. And function *θ*, *ϕ*, *φ* denote three separate 1×1 convolution operations. CAM is optimized by computing the similarity dot product between activations *Y*_*x*,*y*,*z*_ and *Y*_*i*,*j*,*z*_ of *Y* at spatial locations *x*, *y*, *z* and *i*, *j*, *z*, and *g*(*Y*) represents normalization factor. First, redundant convolutional layers and residuals are removed to reduce the number of parameters. Secondly, RSA is applied to the extracted region features. That is, pixel correlation prediction is carried out in the region.

This strategy has several benefits: the region features have a larger field of attention and fewer categories, so the causal interference between pixels can be reduced. It can be beneficial for CAM to have correct coverage of interesting objects. After that, the regional features’ similarity matrix is calculated to weigh the original CAM. We describe this process as follows:

$$\begin{array}{c} {\hat{C}}_{x,y,z}^{*}=\frac{1}{g(\hat{Y})}\sum_{i}^{M}\sum_{j}^{N}f({\hat{Y}}_{x,y,z},{\hat{Y}}_{i,j,z}){\hat{C}}_{x,y,z} \end{array}$$ (4)

$$\begin{array}{c} g(\hat{Y})=\sum_{i}^{M}\sum_{j}^{N}f({\hat{Y}}_{x,y,z},{\hat{Y}}_{i,j,z}) \end{array}$$ (5)

where $C_{x,y,z}^{*}$ is the final modified version of CAM at spatial position *x*, *y*, *z*, and normalized by $g(\hat{Y})$.

$$\begin{array}{c} f\left({\hat{Y}}_{x,y,z}{\hat{Y}}_{i,j,z}\right)=e^{{\hat{Y}}_{x,y,z}^{T}\times {\hat{Y}}_{i,j,z}} \end{array}$$ (6)

in Eq 6, ${\hat{Y}}_{x,y,z}^{T}\times {\hat{Y}}_{i,j,z}$ represents a similar dot product of region features, and the activation function *Relu* is used in $f({\hat{Y}}_{x,y,z},{\hat{Y}}_{i,j,z})$ to mask irrelevant pixels.

After obtaining the region refinement set of CAMs ${\hat{C}}^{*}={\left\{{\hat{c}}_{i}^{*}\right\}}_{i=1}^{Q}$, where ${\hat{c}}_{i}^{*}=\left[{\hat{c}}_{i}^{*(1)},{\hat{c}}_{i}^{*(2)};{\hat{c}}_{i}^{*(3)},{\hat{c}}_{i}^{*(4)}\right]$. ${\hat{C}}^{*}$ is pieced back to the same dimensions as *C* using the Merge module, and we denote this as ${\hat{C}}_{re}^{*}={\left\{{\hat{c}}_{re,i}^{*}\right\}}_{i=1}^{Q}$, where ${\hat{c}}_{re,i}^{*}\in R^{H\times W\times G}$. The global average pooling layer ($h_{\text{GAP}}$) is used to obtain the prediction vector for calculating the classification loss:

$$\begin{array}{c} \left\{ \begin{array}{ccc} \hat{V} & = & h_{\text{GAP}}({\hat{C}}_{re}^{*})\\ V & = & h_{\text{GAP}}(C) \end{array}\right. \end{array}$$ (7)

in Eq 7, where $\hat{V}$ and *V* represents the prediction vector obtained by $C_{re}^{*}$ and *C* through the global average pooling layer. In this study, we only use image-level classification labels to predict the generation of pixel-level pseudo-labels. The multi-label classification loss is used to calculate the classification loss, as shown in Eq 9.

$$\begin{array}{c} l_{cls}\left(X,Y\right)=-\frac{1}{G}\sum_{p=1}^{G}(Y_{p}\;\text{log}\;(\frac{1}{1+e^{-X_{p}}})+(1-Y_{p})\;\text{log}\;(\frac{e^{-X_{p}}}{1+e^{-X_{p}}})) \end{array}$$ (8)

where *G* represents the number of classes. The two steps classification loss is shown in Eq 9:

$$\begin{array}{c} L_{cls}=L_{cls1}+L_{cls2}=l_{cls}(\hat{V},l)+l_{cls}(V,l) \end{array}$$ (9)

where *l* represents the label for classification, this is the only annotation label we use. In the training process, *L*_*cls*_ is simultaneously used as supervision signals for two steps to improve the performance of the classification network.

Through the RSA module, we obtain the refined CAM calculated by the pixel correlation in the region. The refined regional CAM is used as the foreground contrast sample of the CFC module to improve the quality and quantity of the sample to carry out contrastive learning better.

### Cross-image foreground feature comparison module (CFC)

The proposed CFC module, as depicted in Fig 3, aims to enhance the location accuracy of CAMs by leveraging contrastive learning to mine long-range dependencies across different samples. The multiple features $\hat{Y}={\left\{{\hat{y}}_{i}\right\}}_{i=1}^{Q}$ and ${\hat{C}}^{*}={\left\{{\hat{c}}_{i}^{*}\right\}}_{i=1}^{Q}$, where ${\hat{y}}_{i}\in R^{M\times N\times D\times 4}$ and ${\hat{c}}_{i}^{*}\in R^{M\times N\times G\times 4}$, ${\hat{y}}_{i}$ and $c_{i}^{*}$ extracted from step1 are used to construct foreground vector *f*_*i*_ as:

$$\begin{array}{c} f_{i}={\hat{y}}_{i}^{T}\times {\hat{c}}_{i}^{*} \end{array}$$ (10)

where $f_{i}=\left[f_{i}^{\left(1\right)},f_{i}^{\left(2\right)};f_{i}^{\left(3\right)},f_{i}^{\left(4\right)}\right]$. $f_{i}^{\left(j\right)}\in R^{DG\times 1}$. It represents the foreground vector of the image block. We utilize it as a positive samples for contrastive learning. To enhance the network’s robustness to batch size and facilitate its learning process [42], negative sampling is not employed in this case. Then, the rank weights between different sample pairs are calculated by computing the global cosine similarity between foreground vectors in the batch, that is, semantically (appearance, color, or texture) similar pairs are given more weight, and less similar pairs are given less weight, which is used to reduce information confusion between different classes. Different from [30], firstly, we directly apply contrastive learning to multi-channel CAMs, utilizing it as a self-supervisory signal and refining the CAMs through loss calculation during training without introducing additional networks for generating comparison samples. Secondly, our approach is characterized by a dual focus on both global and local similarity, taking into account not only the overall resemblance between images but also the similarities within individual image blocks. $S_{i,r}^{\left(j),(s\right)}$ represents the local similarity matrix between foreground vector matrices $f_{i}^{\left(j\right)}$ and $f_{r}^{\left(s\right)}$:

$$\begin{array}{c} S_{i,r}^{\left(j),(s\right)}=\frac{{\left(f_{i}^{\left(j\right)}\right)}^{T}\times f_{r}^{\left(s\right)}}{\left\|f_{i}^{\left(j\right)}\|\times \|f_{r}^{\left(s\right)}\right\|} \end{array}$$ (11)

then the similarity matrix *S*_*i*,*r*_ between image patches in two different images can be obtained, where $S_{i,r}\in R^{4\times 4}$. This similarity matrix contains the similarity scores corresponding to all locations in *f*_*i*_ and *f*_*r*_. and the global weight between *f*_*i*_ and *f*_*r*_ is defined as:

$$\begin{array}{c} W_{i,r}=\frac{1}{1+e^{-\theta (S_{i,r})}} \end{array}$$ (12)

$$\begin{array}{c} \theta \left(S_{i,r}\right)=\alpha \sum_{x}^{4}\sum_{y}^{4}S_{i,r}\left(x,y\right) \end{array}$$ (13)

in Eq 13, *α* is the weight index used to control function smoothing, *x*, *y* representative location index, and the range of *W*_*i*,*r*_ is between 0 and 1. The cross-image foreground feature vector contrastive loss is shown in Eq 14. It serves as an auxiliary supervision for step 1 generation CAMs. We shrink their feature distance in a self-supervised form during training. To prevent the confusion of different classes of prospects in the training process, *W*_*i*,*j*_ is adopted to calculate the loss:

$$\begin{array}{c} L_{pos}^{B}=-\sum_{i=1}^{b}\sum_{r=1}^{b}I_{i\neq r}(W_{i,r}\;\text{log}(S_{i,r})) \end{array}$$ (14)

where $I_{i\neq r}\in \left\{0,1\right\}$, it is equal to 0 if *i* = *r*. $L_{pos}^{B}$ represents the contrastive loss within batch *B* with batch size *b*, and $L_{pos}=\sum_{B=1}^{U}L_{pos}^{B}$, *U* is the total number of batches. Our ultimate aim is to guide the optimization of the *C* generated in step 2 through the ${\hat{C}}^{*}$ generated in step 1 during training. To make the two steps achieve equivariance learning in the training process, Eq 15 follows the reconstruction regularization proposed by [32, 34], it is reconstruction loss for the original CAM, where ${\hat{C}}_{re}^{*}$ represents the merge version of ${\hat{C}}^{*}$:

$$\begin{array}{c} L_{re}=\|C-{\hat{C}}_{re}^{*}){\|}_{1} \end{array}$$ (15)

the total loss is given in Eq 16, where λ and *β* is the weight coefficient:

$$\begin{array}{c} L=L_{cls}+\lambda L_{pos}+\beta L_{re} \end{array}$$ (16)

During the training process, the model’s parameters are updated in the number of iterations T until the model is fitted, thus expanding the activated region of the CAM to the target actual region. The classifier Λ(⋅) is backpropagated through the gradient to the feature extractor Γ(⋅), we define this part of the parameters as *υ*.

**Algorithm 1**: The training process

**Input**: The training set of images: $X={\left\{x_{i}\right\}}_{i=1}^{Q}$

**Output**: The set of CAMs: $C={\left\{c_{i}\right\}}_{i=1}^{Q}$

**1 for** *t* ← 1 to *T* **do**

**2** $\hat{X}={\left\{{\hat{x}}_{i}\right\}}_{i=1}^{Q}$ ← Chunk(X);

**3** $\hat{Y}={\left\{{\hat{y}}_{i}\right\}}_{i=1}^{Q}\leftarrow \Gamma (\hat{X})$ , $Y={\left\{y_{i}\right\}}_{i=1}^{Q}\leftarrow \Gamma (X)$;

**4** $\hat{C}={\left\{{\hat{c}}_{i}\right\}}_{i=1}^{Q}\leftarrow \Lambda (\hat{Y}),C={\left\{c_{i}\right\}}_{i=1}^{Q}\leftarrow \Lambda (Y)$ ;

**5** ${\hat{C}}^{*}$ ← Extend $\hat{C}$ via Eq 4;

**6**  Extend ${\hat{C}}^{*}$ via Eq 14;

**7** ${\hat{C}}_{re}^{*}={\left\{{\hat{c}}_{re,i}\right\}}_{i=1}^{Q}\leftarrow \text{Merge}({\hat{C}}^{*})$ ;

**8**  Extend *C* via Eq 15;

**9**  Update *υ* via Eq 16;

**10 end**

for testing, given a testing set of images is defined as $K={\left\{ki\right\}}_{i=1}^{R}$. We used steps without modules to generate CAMs for each images.

**Algorithm 2**: The testing process

**Input**: The testing set of images: $K={\left\{k_{i}\right\}}_{i=1}^{R}$

**Output**: The set of CAMs: $C={\left\{c_{i}\right\}}_{i=1}^{R}$

**1** Initialize: The parameters *υ* of Λ(⋅) and Γ(⋅) are loaded using the pre-trained model;

**2** $Y={\left\{y_{i}\right\}}_{i=1}^{R}\leftarrow \Gamma (X)$ ;

**3** $C={\left\{c_{i}\right\}}_{i=1}^{R}\leftarrow \Lambda (Y)$

According to the above steps, we obtain a CAM generative model trained by source samples and image-level classification labels, after which the conventional two post-processing steps are followed: (1) CAM regions are selected as seed regions by threshold [11]. (2) Expand it as the final pseudo-label [18]. And its visualization results are shown in Experimental Results and Disscusion. From the method structure, it is easy to see that the RSA module and CFC module complement each other, the RSA module provides more abundant high-quality samples for the CFC module, and CFC guides the generation of CAM of the RSA module through loss, which we will also prove in our experiments.

## Experimental results and disscusion

### Implementation details

#### Datasets

PASCAL VOC 2012 [43] is currently the most widely used natural scene image data set in weakly supervised image semantic segmentation based on image-level labels. In the weakly supervised semantic segmentation task, use image-level labels for pseudo mask generation and pixel-level labels for validating semantic segmentation results—training using an enhanced training set of 10,582 images, 1,449 for validation, and 1,456 for testing. MS COCO 2014 dataset [44] consists of 80classes,with 82,783 and 40,504 images for training and validation. In all experiments, the image is randomly scaled in the range of [320, 640] and then clipped to 512 × 512 as the network input.

#### Evaluation index

The mean Intersection over Union (mIoU) [45] was used as the overall performance evaluation index of the experiment’s pseudo-label generation end and segmentation end. The calculation formula is as follows:

$$\begin{array}{c} mIoU=\frac{1}{G}\sum_{p=1}^{G}\frac{TP_{p}}{FN_{p}+FP_{p}+TP_{p}} \end{array}$$ (17)

mean false discovery rate (mFDR) and mean false negative rate (mFNR) are used as the CAM’s prediction performance evaluation index. Specifically when the CAM can cover more object target areas, the value of mFNR will be smaller. When the false activations of CAM are less, the mFDR is smaller [19, 32]:

$$\begin{array}{c} mFDR=\frac{1}{G}\sum_{p=1}^{G}\frac{FP_{p}}{FP_{p}+TP_{p}} \end{array}$$ (18)

$$\begin{array}{c} mFNR=\frac{1}{G}\sum_{p=1}^{G}\frac{FN_{p}}{FN_{p}+TP_{p}} \end{array}$$ (19)

where *TP*_*p*_ denotes the pixel number of accurate positive prediction of class p; *FP*_*p*_ and *FN*_*p*_ indicate the number of false positive and false negative predictions of class *p*.

### Comparison experiment

#### Training details

In this study, experimental hardware equipment is CPU: 15 vCPU Intel(R) Xeon(R) Platinum 8358P CPU @ 2.60GHz; GPU: A100-SXM4-80GB(80GB) * 1. The initial learning rate of the generator is 0.01, the batch size is 32, and the maximum iteration number T is 4.5k. The comparative experiment of pseudo-label quality was performed with the previous methods, all of which were performed on the voc2012 dataset.

The proposed method uses PuzzleCam as baseline and analyzes two backbone networks, resnet50 and resnest101. According to the verification on the voc2012 training dataset, Table 1 shows the mIoU of the CAMs generated by the proposed method for different combinations of λ and *β* values. Where *L*_*re*_ learns the difference between the regions of interest of the full and segmented images, and *L*_*pos*_ brings the feature representations of similar foreground classes closer together during training, during the training process, we found that *L*_*pos*_ is more sensitive than *L*_*re*_, so we set a minor parameter change for *L*_*pos*_ to select an appropriate λ, and *α* = 0.25. As can be seen from the table, when λ = 0.5 and *β* = 2, the generated CAMs achieve the highest mIoU, and the subsequent experiments are also carried out under the parameter setting.

**Table 1:** Comparative experiments based on different values of λ and *β*, the quality of CAMs assessed by PASCAL VOC2012 training dataset in mIoU%.

*β*	1	2	3
λ			
0.5	54.87	**56.75**	56.07
1	53.63	54.32	54.98
1.5	47.26	49.82	51.13

#### Comparison with baseline

The quality of pseudo-labels determines the performance of weakly supervised semantic segmentation networks. Table 2 shows that Under different backbone conditions after adding the two modules RSA and CFC proposed in this paper, the pseudo-labels generated by the proposed method increase by 5.22 and 3.43 relative to PuzzleCam and proves that our semantic information mining network effectively improves pseudo-label quality.

**Table 2:** Comparison experiment with PuzzleCam: Two different backbones are used to experiment on PASCAL VOC2012 training dataset in mIoU%.

Mothod	backbone	CAM(%)
PuzzleCam [34]	ResNet-50	51.53
PuzzleCam [34]	ResNeSt-101	61.85
Ours	ResNet-50	**56.75**
Ours	ResNeSt-101	**65.28**

**Fig 4:**
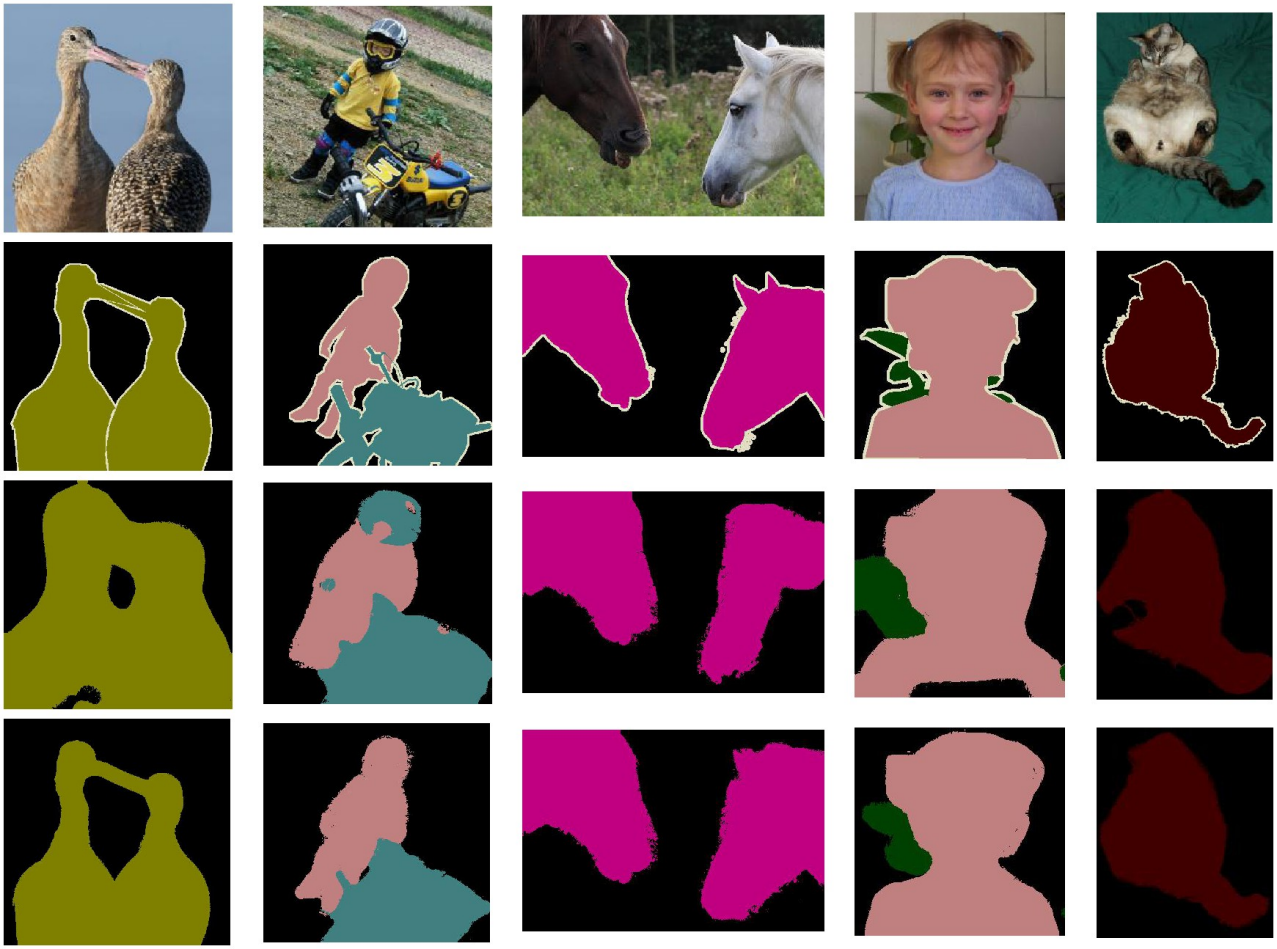
Pseudo masks on PASCAL VOC 2012 train dataset. From top to bottom are original images; ground truth; The prediction results of PuzzleCam; The prediction results of our method.

The results of pseudo-labels visualization are shown in Fig 4. It can be seen that when there is single or multi-class information in the scene, the pseudo-labels generated by us have a more accurate prediction range. Taking the first column as an example, through the self-supervised signal introduced by our CFC module, ours has a more accurate prediction of the foreground and background compared to PuzzleCam. For example, in PASCAL VOC2012, because persons and motorcycles often appear in the same scene, PuzzleCam will predict the false correlation between them, resulting in a boundary range that is difficult to distinguish between different classes. Our approach benefits from guiding the activation of the CAM by pixel correlations at a regional scale, effectively reducing the causal intervention of inter-pixel errors.

**Fig 5:**
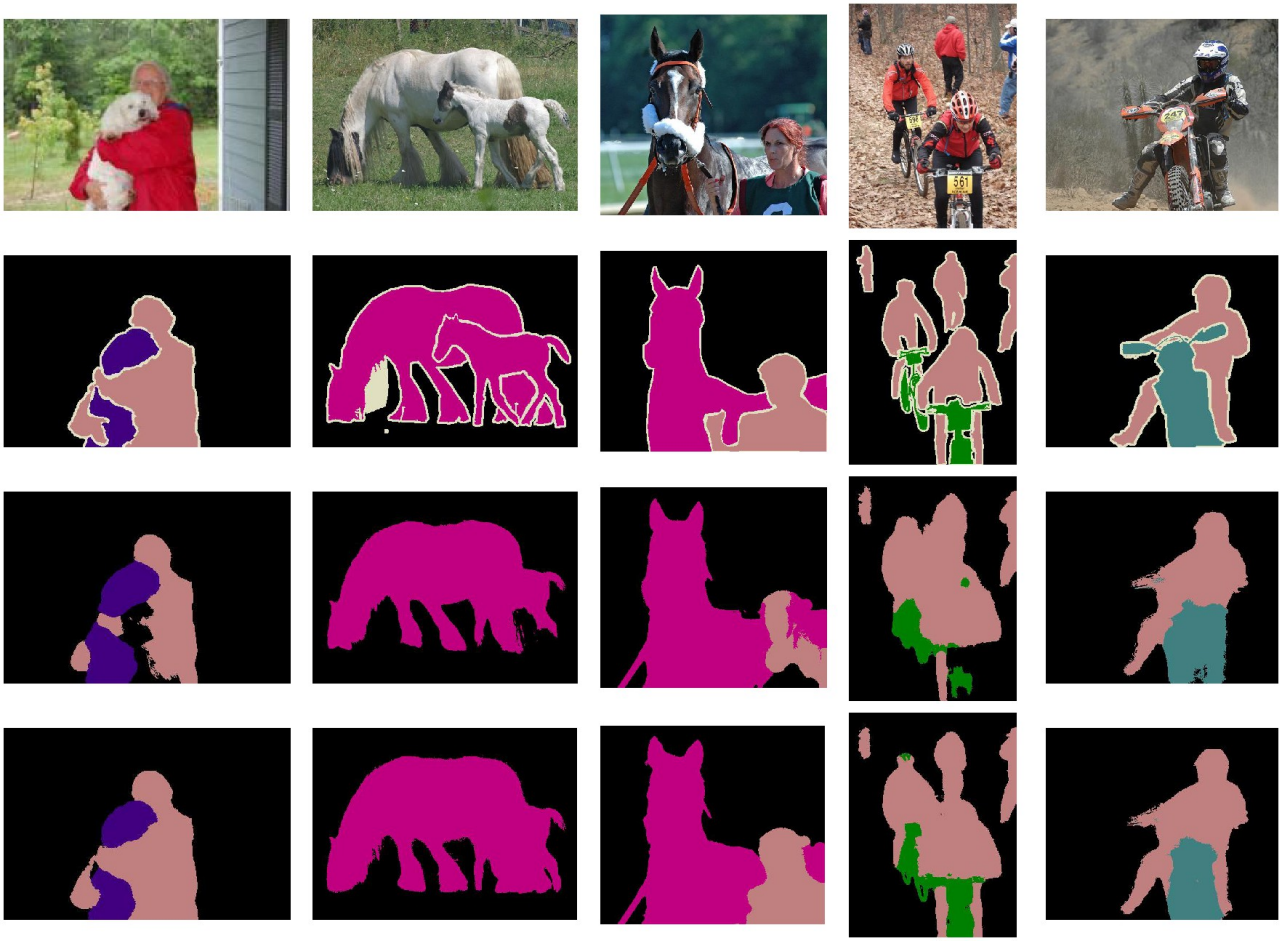
Pseudo masks on PASCAL VOC 2012 val dataset. From top to bottom are original images; ground truth; The prediction results of PuzzleCam; The prediction results of our method.

The segmentation result is one of the criteria to measure the quality of the pseudo-label. To further prove the effectiveness of the proposed method, we use the pseudo-labels generated by the method based on resnest101 in this paper to train DeeplabV3+ [36], the segmentation results on PASCAL VOC2012 validation set are shown in Fig 5. With the same segmentation end, Fig 5 shows that our DeeplabV3+ achieves high-quality segmentation results in different scenarios even though we do not use any saliency label supervision during training, especially in complex scenes, the results of PuzzleCam often fall into misjudgment in some ambiguous regions. For example, our segmentation results can accurately determine the boundary range of different classes in the overlapping regions of person and dog. When multiple targets are in the scene, it can locate their positions more accurately. The gap between the target range predicted by the segmentation network and the ground truth is smaller.

#### Comparison with mainstream method

In order to analyze the superiority of our method more clearly, the prediction accuracy of each class and background is compared with other mainstream methods in mIoU on the PASCAL VOC 2012 val dataset. As seen from Table 3, the table’s highest value is in bold. For some easily confused classes, such as bicycles, horses, and motorcycles, which often appear in the same scene with people, our method achieves more accurate prediction results, which is essentially due to the correct long-range dependencies learned by the network through our method. However, for example, birds and ships, which are usually small in area, are difficult to match with foreground classes with higher similarity in the batch size after blocking, so our prediction results have low accuracy.

**Table 3:** Performance on the PASCAL VOC 2012 validation set, compared to weakly supervised approaches based only on image-level labels.

**Method**	**bkg**	**aero**	**bike**	**bird**	**boat**	**bottle**	**bus**	**car**	**cat**	**chair**	**cow**
SEC [10]	82.4	62.9	26.4	61.6	27.6	38.1	66.6	62.7	75.2	22.1	53.5
MCOF [36]	85.8	**74.1**	23.6	66.4	36.6	62.0	75.5	68.5	78.2	18.8	64.6
AffinityNet [19]	**88.2**	68.2	30.6	**81.1**	**49.6**	61.0	77.8	66.1	75.1	29.0	66.0
Ours	86.7	72.5	**33.8**	43.1	28.7	**73.1**	**85.6**	**78.9**	**88.6**	**33.5**	**87.2**
**Method**	**table**	**dog**	**horse**	**mbk**	**person**	**plant**	**sheep**	**sofa**	**train**	**tv**	**mean**
SEC [10]	28.3	65.8	57.8	62.3	52.5	32.5	62.6	32.1	45.4	45.3	50.7
MCOF [36]	29.6	72.5	61.6	63.1	55.5	37.7	65.8	32.4	**68.4**	39.9	56.2
AffinityNet [19]	40.2	80.4	62.0	70.4	73.7	42.5	70.7	42.6	68.1	**51.6**	61.7
Ours	**55.5**	**87.0**	**78.9**	**76.1**	**80.8**	**51.6**	76.4	**51.0**	49.6	43.7	**64.9**

The proposed method is compared with other methods on the PASCAL VOC 2012 test datasets. As seen from Table 4, the segmentation model trained by our pseudo-labels also achieved superior results and excellent generalization ability on the test set. Among them, SEC [10] and AffinityNet [19] also improve the prediction of cam through the context relationship between pixels, and our segmentation results are improved by 11.8% and 2.1%, respectively. And different from AffinityNet [19], the method of generating CAM in this paper is end-to-end, and no other network is used to refine the CAM further.

**Table 4:** Performance on the PASCAL VOC 2012 test set, compared to weakly supervised approaches based only on image-level labels.

**Method**	**bkg**	**aero**	**bike**	**bird**	**boat**	**bottle**	**bus**	**car**	**cat**	**chair**	**cow**
SEC [10]	83.5	56.4	28.5	64.1	23.6	46.5	70.6	58.6	71.3	23.2	54.0
MCOF [36]	87.0	71.6	26.1	63.9	32.6	57.5	75.7	68.2	75.0	19.9	67.6
AffinityNet [19]	**89.1**	70.6	31.6	**77.2**	42.2	**68.9**	**79.1**	66.5	74.9	**29.6**	68.7
Ours	87.7	**71.8**	**49.8**	68.1	**48.8**	58.9	68.2	**74.5**	**88.3**	29.1	**90.5**
**Method**	**table**	**dog**	**horse**	**mbk**	**person**	**plant**	**sheep**	**sofa**	**train**	**tv**	**mean**
SEC [10]	28.0	68.1	62.1	70.0	55.0	38.4	58.0	39.9	38.4	48.3	51.7
MCOF [36]	36.1	74.6	69.9	76.5	55.9	42.6	73.8	33.5	65.2	41.0	57.8
AffinityNet [19]	56.1	**82.1**	64.8	78.6	73.5	**50.8**	70.7	**47.7**	63.9	**51.1**	63.7
Ours	**63.5**	71.3	**74.1**	**80.1**	**79.8**	50.4	**88.3**	22.6	**65.4**	49.4	**65.8**

We compare our trained DeeplabV3+ with other current mainstream semantic segmentation methods trained on image-level labels and saliency labels. For a fair comparison,and follow the established process used in previous work, Random Walk (RW) [19], dense conditional random field (CRF) [46] are used in this experiment to refine the generated pseudo label further. Table 5 shows that our proposed method achieves the highest mIoU on both the val set and the test set of PASCAL VOC2012. Among them, CIAN [33] is also used for long-range dependencies learning across images. Compared with CIAN [33], the proposed method improves the validation set and test set by 0.6% and 0.5%, respectively. Compared with MDC [25], the proposed method improves the mIoU by 4.5% and 5.0% on the val and test sets, respectively, with weaker annotations.

**Table 5:** Comparison of our proposed method and existing state-of-the-art methods on the PASCALVOC2012 val and test. I, image-level labels; S, saliency label.

Mothod	backbone	Supervision	val	test
MDC [25]	ResNet-101	I+S	60.4	60.8
DSRG [17]	ResNet-101	I+S	61.4	63.2
SeeNet [47]	VGG16	I+S	63.1	62.8
EM-Adapt [48]	VGG16	I	38.2	39.6
MIL-LSE [14]	Overfeat	I	42.0	40.6
CRF [46]	VGG16	I	52.8	53.7
RRM [49]	ResNet-38	I	62.6	62.9
IRNET [11]	ResNet-50	I	63.5	64.8
IAL [36]	ResNet-38	I	64.3	65.4
GWSM [21]	VGG16	I	63.3	63.6
SSDD [50]	ResNet-38	I	64.9	65.5
CIAN [33]	VGG16	I	64.3	65.3
AMN [51]	ResNet-101	I	69.5	69.6
URN [52]	ResNet-101	I	69.5	69.7
RS [53]	ResNet-50	I	75.2	76.7
SANCE [54]	ResNet-101	I	76.9	70.9
MARS [55]	ResNet-101	I	**77.7**	77.2
Ours	ResNest-101	I	64.9	65.8
Ours+RW [19] + CRF [46]	ResNest-101	I	76.6	**77.4**

We further evaluate the performance of our model on MS COCO 2014, where pixel-level annotations are available, We solely utilized image-level class labels during the training procedure. It should be noted that in order to reduce computational costs, we have opted to train on a subset of the training images, specifically 50% (40k) images. Experimental Results Table 6 compares our approach and current WSSS methods with image-level supervision on the COCO dataset. We can observe that our method achieves mIoU score of 34.2% on the val set, outperforming all the competitors.

**Table 6:** Comparison of our proposed method and existing methods on the MS COCO 2014 val.

**Mothod**	**backbone**	**mIoU(%)**
SEC [10]	VGG16	22.4
DSRG [17]	ResNet-101	26.0
IAL [36]	ResNet-38	27.7
SEAM [32]	ResNet-38	31.9
IRNET [11]	ResNet-50	32.6
AMN [51]	ResNet-101	**44.7**
URN [52]	ResNet-101	40.7
Ours	ResNest-101	34.2
Ours+RW [19] + CRF [46]	ResNest-101	43.8

The above visual experimental results show that the proposed pseudo-label generation method has a more accurate region mask than similar methods. The segmentation network trained with our pseudo-labels achieves the highest prediction accuracy, effectively narrowing the gap between weakly supervised and fully supervised methods.

### Ablation experiment

To verify the independent validity of the two modules, ResNet50 and ResNset101 were used as the backbone network for analysis. As seen from Table 7, when the CFC module is added to the baseline, the mIoU of the pseudo-label is improved by 2.57%. When the RSA module is added to the baseline, the mIoU of the pseudo-label is improved by 4.45%. The best mIoU is achieved when the two modules are used in parallel, with 5.22% improvement over baseline. The experimental results show that the two modules proposed in this paper effectively improve the pseudo-label quality, and the effect is best when combined.

**Table 7:** Ablation experiment of mIoU. The quality of the pseudo mask is evaluated on PASCAL VOC2012 Training Dataset. ResNet50 was used as the backbone.

baseline	CFC	RSA	mIoU(%)
√			51.53
√	√		54.10
√		√	55.98
√	√	√	**56.75**

**Fig 6:**
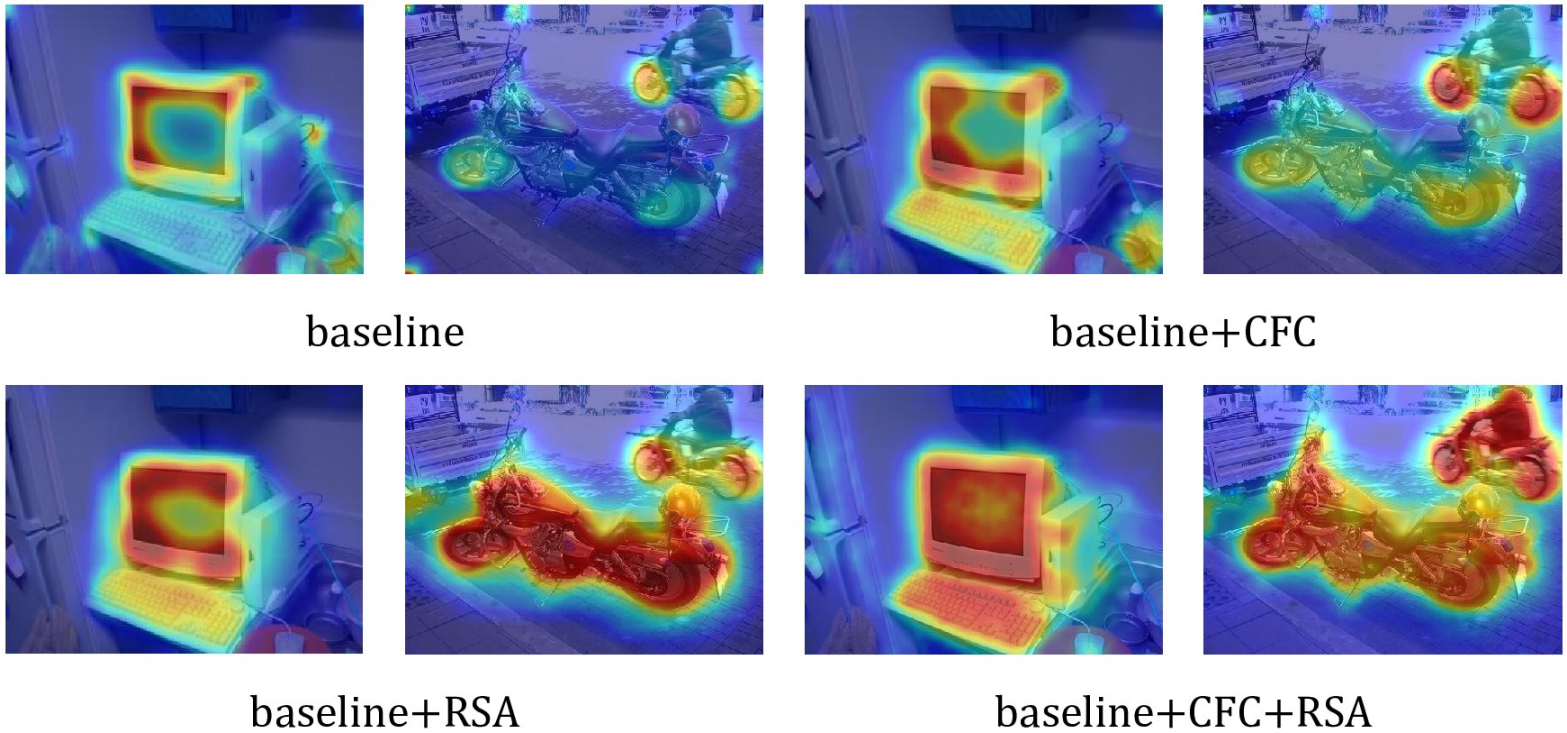
Ablation experiment of CAM. ResNet50 was used as the backbone network for analysis. CAM in the figure is generated by PASCAL VOC2012 Training Dataset.

We visualize the CAM effects achieved by combining different modules. Fig 6 shows CAMs generated by the different modules, and the CAMs shown are the set of all class predictions. It can be seen that baseline-based CAMs tend to be limited to regions of salient features of objects, such as the wheels of a motorcycle. When the CFC module is added, the prediction region of CAMs expands from the salient regions of the target to other regions. When the RSA module is added, the false activation area of CAMs is visibly reduced. Cams have richer detail and more accurate activation when CFC and RSA are used together. This benefits from more reliable pixel semantic information mined through the two modules designed in this paper.

**Fig 7:**
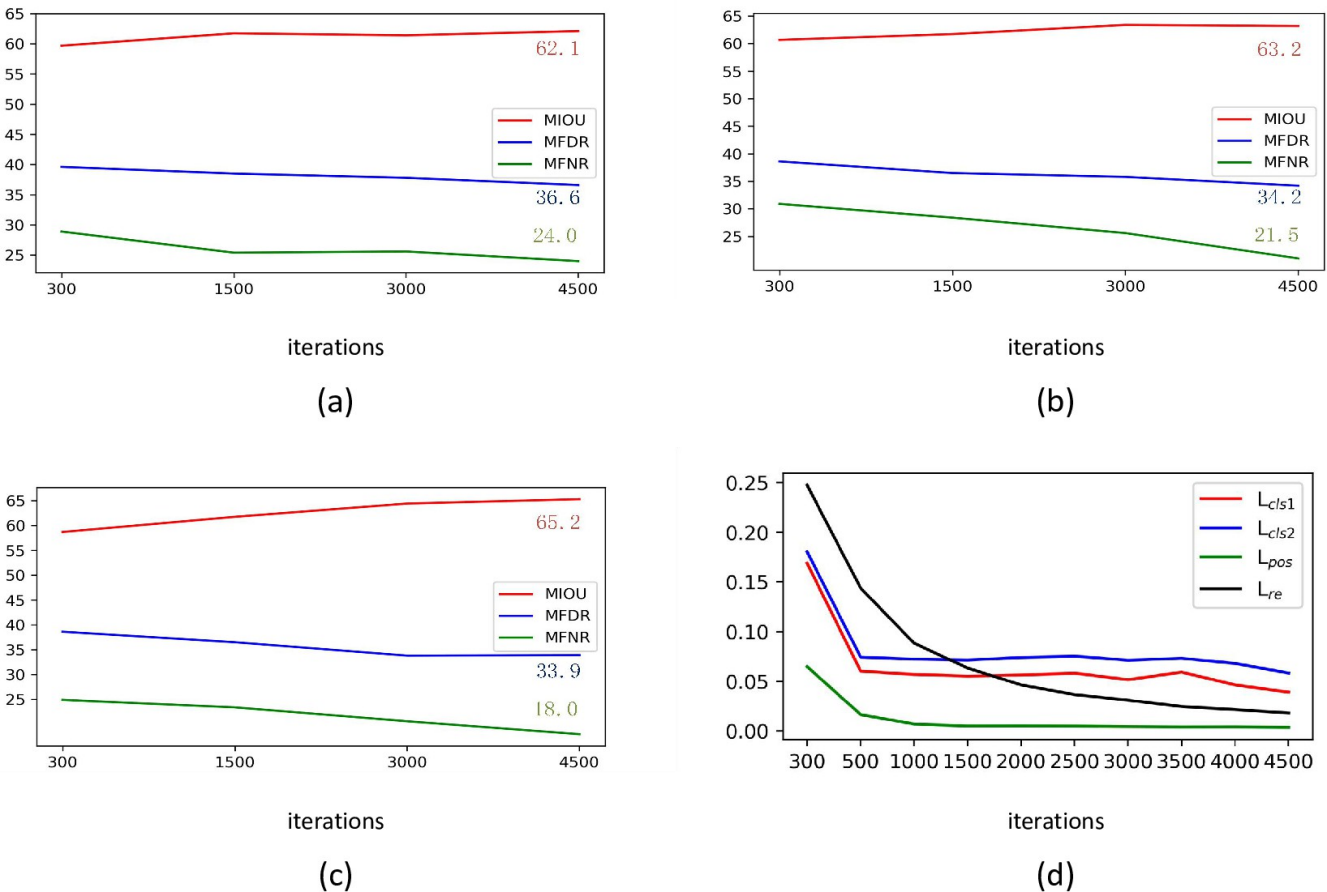
Ablation on the iterations number of PASCAL VOC2012 Training Dataset, and shows the mIoU, mFDR, and mFNR for different iterations, the backbone network is ResNest-101: (a) *baseline* + *CFC*; (b) *baseline* + *RSA*; (c) *baseline* + *CFC* + *RSA*; (d) shows the loss for the different number of iterations of the final version (c).

To better understand how our method can effectively mine out more pixel-level semantic information, we sample the generated CAM at different iterations. Fig 7 visualizes the decrease in loss and the improvement in model accuracy as the number of iterations increases during training. When the CFC and RSA modules are added using the same backbone, the experimental results show that the pseudo-label achieves lower mFDR and mFNR, indicating that the generated CAMs cover more target area and fewer false predictions. At the same time, the higher mIoU means that the pseudo-label has higher overall prediction accuracy. Combining the two modules is beneficial to improve the quality of pseudo-labels further. The main performance gains come from the effectiveness of CFC and RSA and the cooperation of CFC and RSA, in which the correct long-range dependencies are learned from intra-sample and inter-sample.

**Fig 8:**
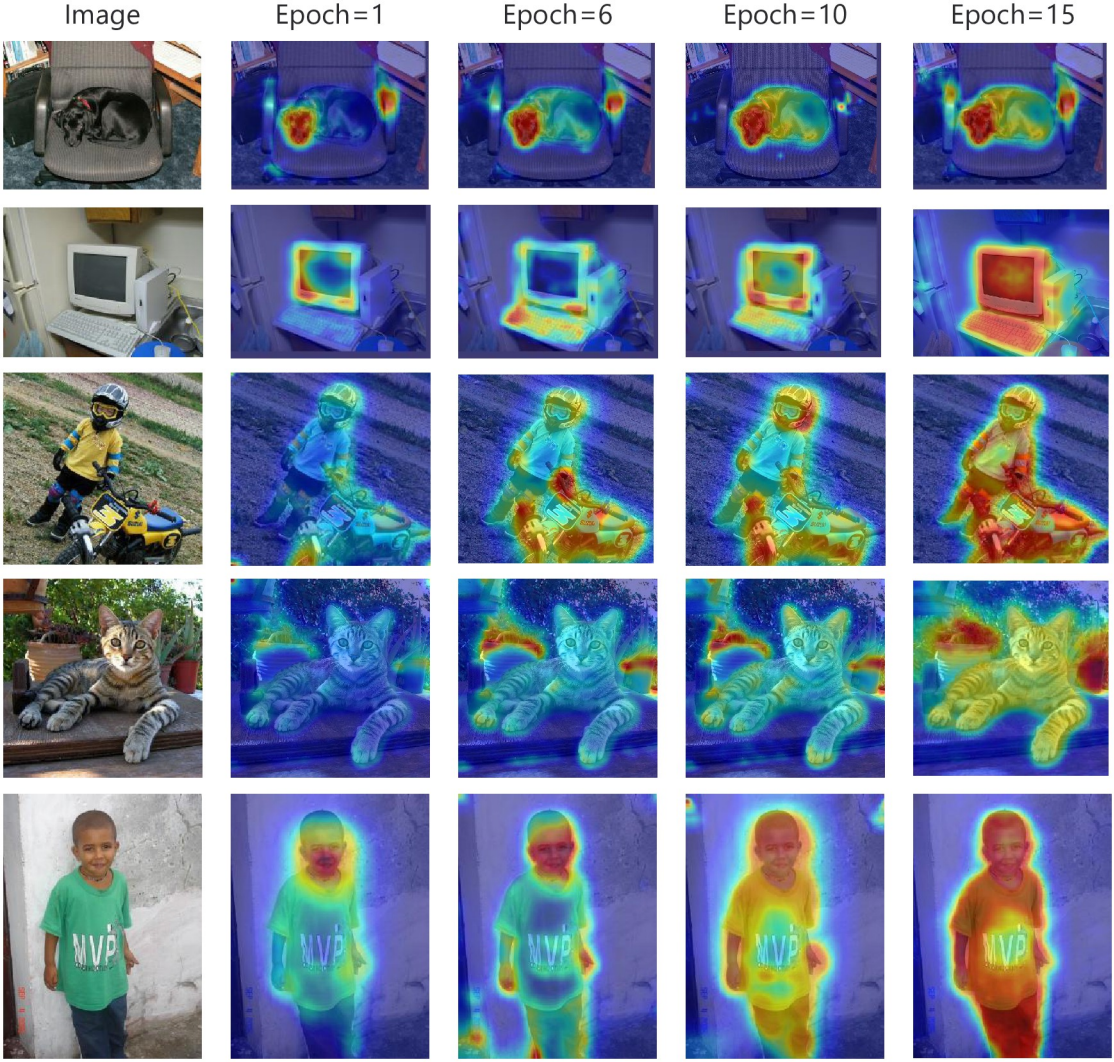
Column 1 is the original figure, and columns 1, 3, 4, and 5 represent the cam generated with different training epoch.

Fig 8 visualizes the CAMs generated by the final version(c) under different training rounds. It can be seen that with the increase of training rounds, through the supervision information we add, the activation area of CAMs is effectively extended from the salient local area of the target to other regions. The CAMs have smoother and more complete boundaries, some targets normally ignored by the network, such as the human body and chair, were also activated after training.

The above ablation experiments show that the two core modules in the proposed framework effectively improve the quality of pseudo-labels generated based on image-level labels when evaluated separately. When the two modules are combined, higher pseudo-label accuracy is achieved. The effectiveness of the additional supervision incorporated in this paper is demonstrated by visualizing the accuracy and expansion trend of CAMs during training. It reduces the CAM fragmentation activation problem caused by the classification tasks. And the overall experiment shows that through two modules embedded in classification network, we successfully mined richer semantic information and greatly improved the executable of weakly supervised learning.

## Conclusions

In this paper, a novel weakly supervised semantic segmentation framework is proposed. We extend the CAMs generated by the classification network, using the long-range dependencies. We propose the cross-image foreground feature contrast module and the regional self-attention module, which take into account both inter-sample relationships and information confusion arising from such dependencies. The results demonstrate that these two modules effectively extract more semantic information and accurate target range regions, resulting in a CAM with expanded coverage over the entire target area and fewer false predictions. The method enhances the precision of pseudo-labels for semantic segmentation networks. However, there is a need to improve the accuracy of small object detection when generating pseudo labels.
